# Supplementary material for: Social Chemical Communication Determines Recovery From L1 Arrest via DAF-16 Activation
Source: Front Cell Dev Biol. 2020 Nov 10;8:588686. doi: 10.3389/fcell.2020.588686 (PMC7683423; doi:10.3389/fcell.2020.588686)
Supplement: FIGURE S1 — Developmental time (M1–M4) (A) and complete the post-embryonic development (L1–M4) (B) of animals arrested at densities of 20, 2, and 0.2 L1/μl, after 1, 4, 8, and 14 days of starvation. All individual animals from three independent biological replicates are plotted. Black dots represent the average of each independent replicate and the black line indicates the mean of the entire experiment. Averages of the replicates were used for statistics (∗p < 0.05, unpaired t-test). (C) Subcellular localization of DAF-16::GFP in L1 larvae arrested at densities of 20, 2, and 0.2 L1/μl for 1 to 5 days in starvation. Dots indicate the mean of four independent replicates and the error bars represent the SEM (∗p < 0.05, ∗∗p < 0.01, ∗∗∗p < 0.001, one-way ANOVA). [file Data_Sheet_1.pdf]

Supplementary material for:

Social chemical communication determines recovery from L1 arrest via DAF-16 activation.

Alejandro Mata-Cabana[1], Laura Gómez-Delgado[1], Francisco Javier Romero-Expósito[1], María Jesús Rodríguez-Palero[2], Marta Artal-Sanz[2] and María Olmedo[1]

1. Departamento de Genética, Facultad de Biología, Universidad de Sevilla, Avenida Reina Mercedes s/n, 41012 Seville, Spain

2. Andalusian Center for Developmental Biology, Consejo Superior de Investigaciones Científicas/Junta de Andalucía/Universidad Pablo de Olavide. Department of Molecular Biology and Biochemical Engineering. Carretera de Utrera Km 1, 41013 Seville, Spain.

Correspondence: amata@us.es, mariaolmedo@us.es

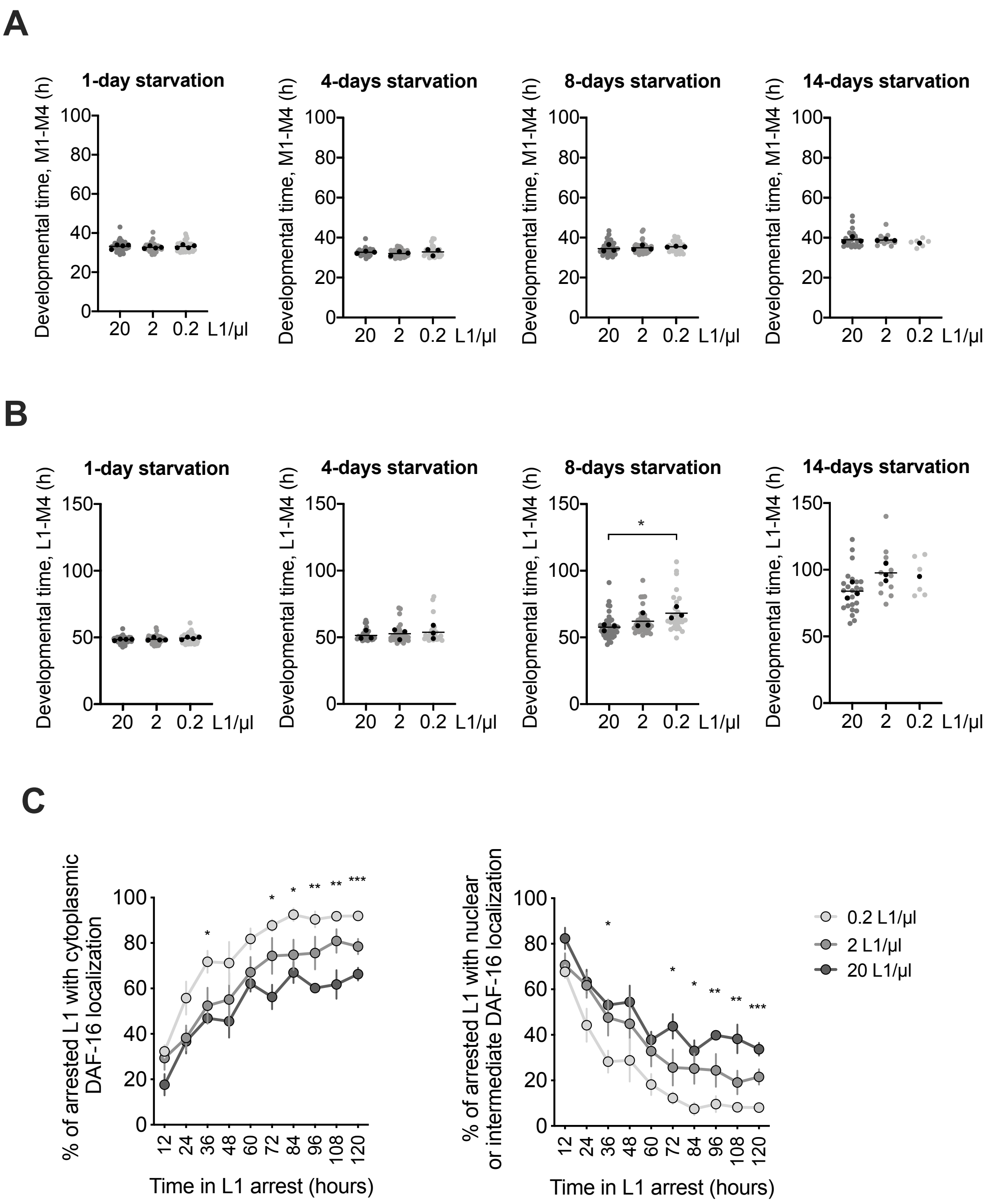

Supplementary Figure S1. Developmental time (M1 to M4) (A) and complete the post-embryonic development (L1 to M4) (B) of animals arrested at densities of 20, 2 and 0.2 L1/μl, after 1, 4, 8 and 14 days of starvation. All individual animals from three independent biological replicates are plotted. Black dots represent the average of each independent replicate and the black line indicates the mean of the entire experiment. Averages of the replicates were used for statistics (* $p<0.05$, Unpaired t-test). (C) Subcellular localization of DAF-16::GFP in L1 larvae arrested at densities of 20, 2 and 0.2 L1/μl for 1 to 5 days in starvation. Dots indicate the mean of four independent replicates and the error bars represent the SEM (* $p<0.05$, ** $p<0.01$, *** $p<0.001$, one-way ANOVA).

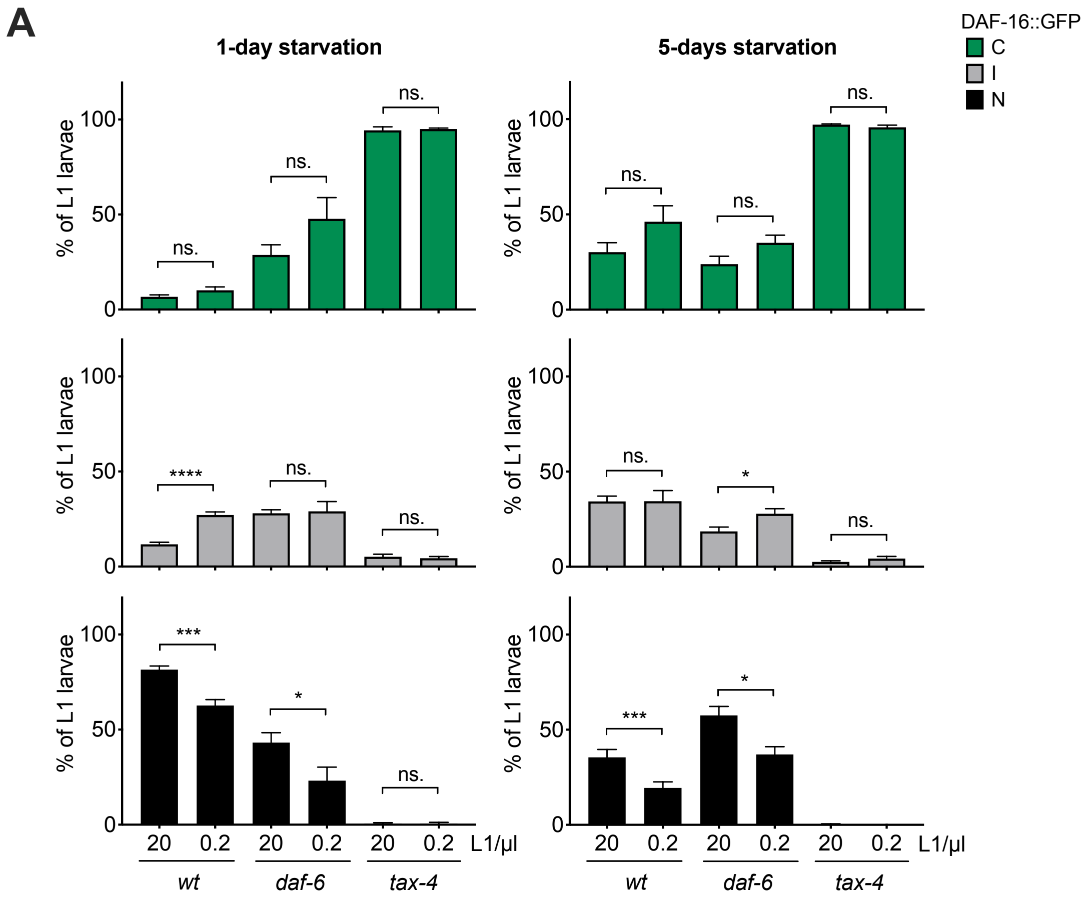

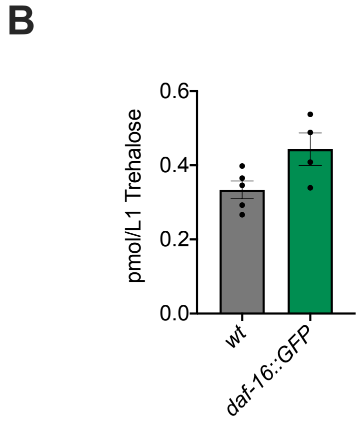

Supplementary Figure S2. (A) Subcellular localization of DAF-16::GFP in L1 larvae arrested at 20 and 0.2 L1/µl for 1 and 5 days in *wt*, *daf-6* and *tax-4* mutant strains. Percentage of animals with cytoplasmatic localization are shown in the top panel, percentage of animals with intermediate localization are in the middle panel and nuclear localization of DAF-16 is shown in the bottom panel. The histograms show the means of, at least, three independent replicates and the error bars represent the SEM (* p<0.05, *** p<0.001, **** p<0.0001, Unpaired t-test). (B) Relative trehalose content per hatched animal in the medium of wild-type and TJ356 reporter strain, which expresses DAF-16::GFP.

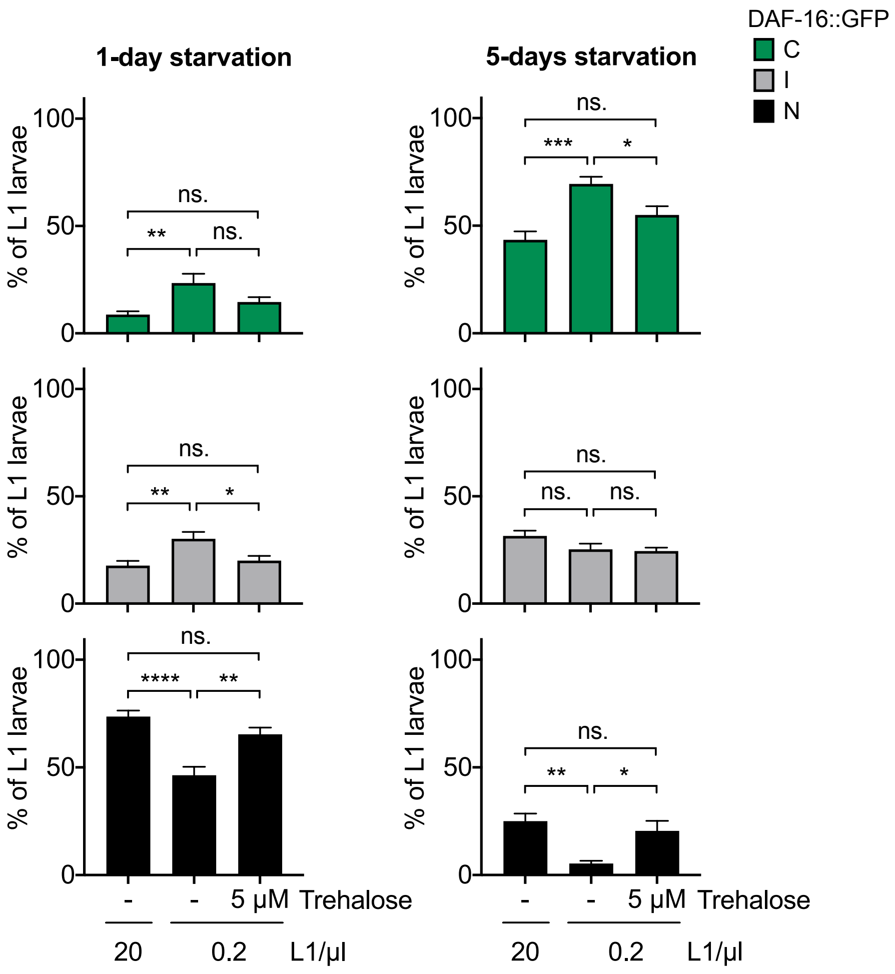

Supplementary Figure S3. Effect on the subcellular localization of DAF-16::GFP of the addition of 5 μM of trehalose to L1 larvae arrested at 0.2 L1/μl after 1 and 5 days of starvation when compared with animals arrested at 20 and 0.2 L1/μl with no trehalose addition. Percentage of animals with cytoplasmatic localization are shown in the top panel, percentage of animals with intermediate localization are in the middle panel and nuclear localization of DAF-16 is shown in the bottom panel. The histograms show the means of eight independent replicates and the error bars represent the SEM (* p<0.05, ** p<0.01, **** p<0.0001, one-way ANOVA).

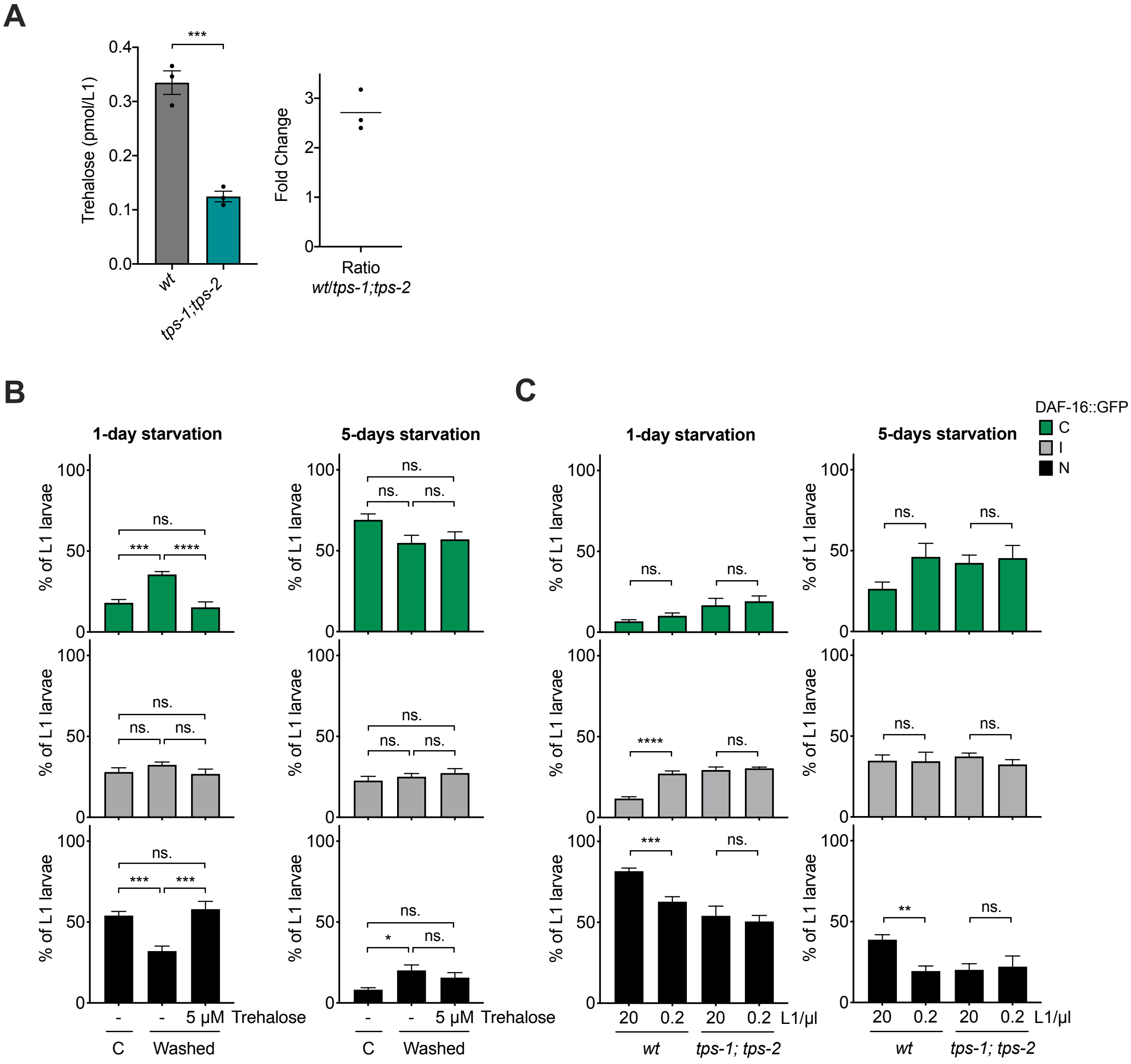

Supplementary Figure S4. (A) In the left panel, relative trehalose content per hatched animal in the medium of wild-type and *tps-1; tps-2* strains. Black dots represent the average of each independent replicate. The bars mark the mean of the entire experiment and error bars shows the SEM. Averages of each replicate were used for statistics (*** $p<0.001$, Unpaired t-test). In the right panel, trehalose content ratio between wild type and *tps-1; tps-2* independently calculated for each replicate. The horizontal line indicates the mean of the three replicates. (B) Subcellular localization of DAF-16::GFP in L1 larvae arrested at 20 L1/μl at 1 and 5 days after washing the medium. Trehalose was added to one sample to a final concentration of 5 μM. In control sample the original medium was not removed and trehalose was not added. (C) Subcellular localization of DAF-16::GFP in wild-type and *tps-1; tps-2* L1 larvae arrested at 20 and 0.2 L1/μl, after 1 and 5 days of starvation. (C and D) Percentage of animals with cytoplasmatic localization are shown in the top panel, percentage of animals with intermediate localization are in the middle panel and nuclear localization of DAF-16 is shown in the bottom panel. The histograms show the means of, at least, four independent replicates and the error bars represent the SEM (* $p<0.05$, ** $p<0.01$, *** $p<0.001$, **** $p<0.0001$, one-way ANOVA in (C) and Unpaired t-test in (D)).

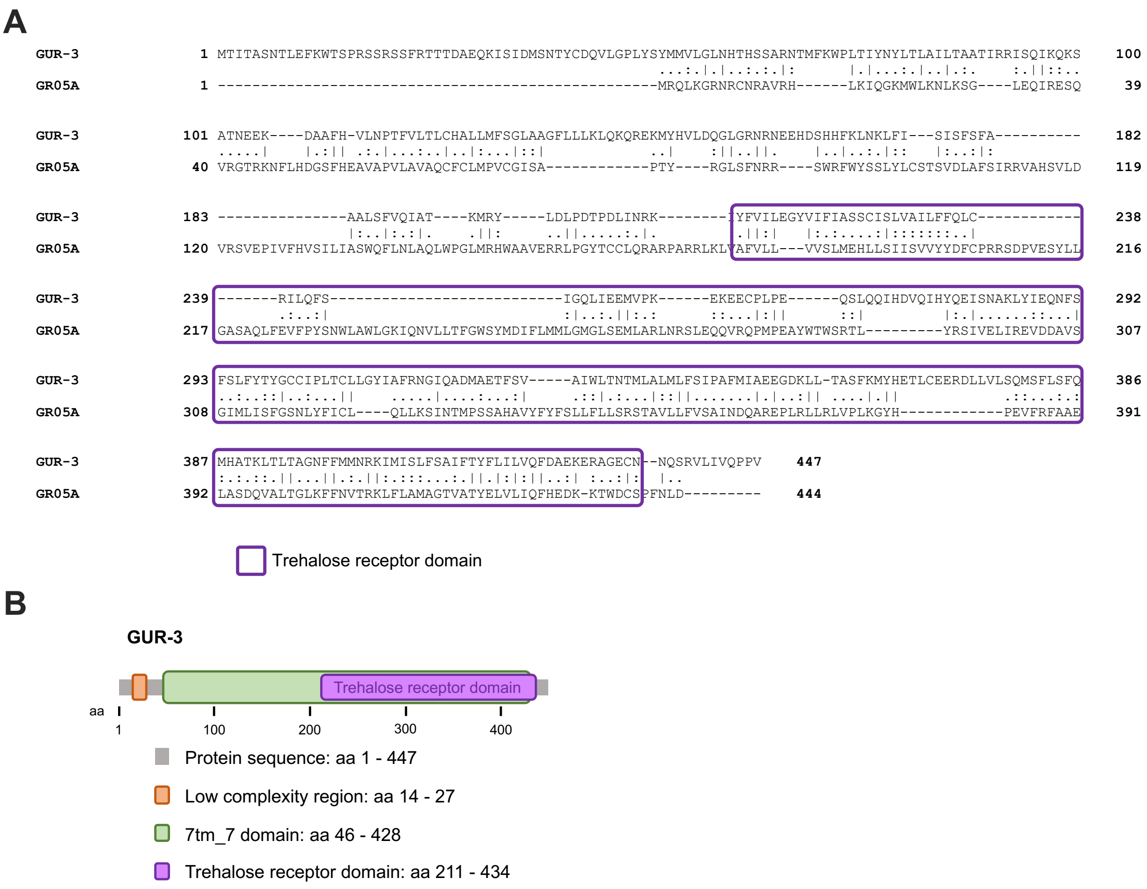

Supplementary Figure S5. (A) Pairwise protein sequence alignment between GUR-3 and Gr5a using EMBOSS-Needle tool (https://www.ebi.ac.uk/Tools/psa/emboss_needle/). Predicted trehalose receptor domain is framed in purple. (B) Schematic representation of GUR-3 protein domains. Prediction was performed using SMART on-line tool (http://smart.embl-heidelberg.de/).
